# Accelerating the development of implantable neurochemical biosensors by using existing clinically applied depth electrodes

**DOI:** 10.1007/s00216-022-04445-1

**Published:** 2022-12-02

**Authors:** Alexander R. Macdonald, Francessca Charlton, Damion K. Corrigan

**Affiliations:** 1 Department of Biomedical Engineering, University of Strathclyde, 106 Rottenrow East, Glasgow, UK; 2 Department of Pure and Applied Chemistry, University of Strathclyde, 295 Cathedral Street, Glasgow, UK

**Keywords:** Enzymes, Amperometric biosensors, In vivo implantation, Neurotransmitters

## Abstract

In this study, an implantable stereo-electroencephalography (sEEG) depth electrode was functionalised with an enzyme coating for enzyme-based biosensing of glucose and L-glutamate. This was done because personalised medicine could benefit from active real-time neurochemical monitoring on small spatial and temporal scales to further understand and treat neurological disorders. To achieve this, the sEEG depth electrode was characterised using cyclic voltammetry (CV), differential pulse voltammetry (DPV), square wave voltammetry (SWV), and electrochemical impedance spectroscopy (EIS) using several electrochemical redox mediators (potassium ferri/ferrocyanide, ruthenium hexamine chloride, and dopamine). To improve performance, the Pt sensors on the sEEG depth electrode were coated with platinum black and a crosslinked gelatin-enzyme film to enable enzymatic biosensing. This characterisation work showed that producing a useable electrode with a good electrochemical response showing the expected behaviour for a platinum electrode was possible. Coating with Pt black improved the sensitivity to H_2_O_2_ over unmodified electrodes and approached that of well-defined Pt macro disc electrodes. Measured current showed good dependence on concentration, and the calibration curves report good sensitivity of 29.65 nA/cm^2^/μM for glucose and 8.05 nA/cm^2^/μM for L-glutamate with a stable, repeatable, and linear response. These findings demonstrate that existing clinical electrode devices can be adapted for combined electrochemical and electrophysiological measurement in patients and obviate the need to develop new electrodes when existing clinically approved devices and the associated knowledge can be reused. This accelerates the time to use and application of in vivo and wearable biosensing for diagnosis, treatment, and personalised medicine.

**Graphical abstract:**

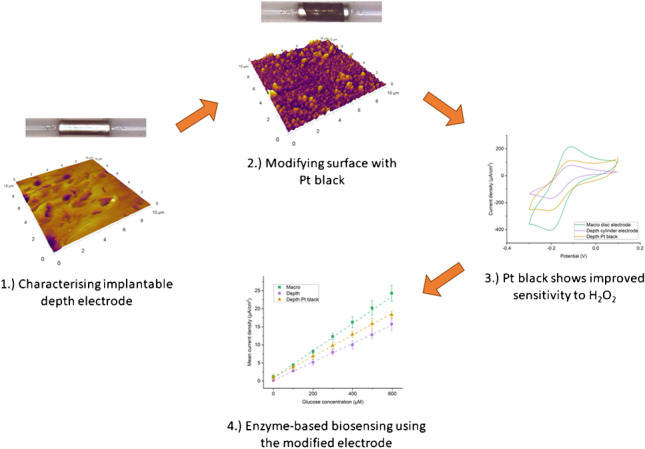

## Introduction

Neurochemical fluctuations are poorly explored compared to electrophysiological measurements for understanding and treating neurological disorders [1, 2]. Researchers, clinicians, and patients could all benefit from improved access to this information. Real-time chemical measurements are challenging without advanced imaging systems or highly invasive sampling methods [3–6]. One area where the ability to combine electrophysiology measurements with electrochemical determination of the levels of different neurotransmitters is for epilepsy where it can be necessary to identify areas of the brain requiring surgical resection [7–10]. Developing a clear picture of the nature of the neurological dysfunction and a well-demarcated picture of the region responsible for seizures can lead to improved identification of areas requiring resection [11].

Many electrodes designed for use in the brain are custom-made, and most are based on the kinds of electrodes used in animal research such as carbon fibre microelectrodes and silicon shank–based devices [12–15]. Implantable electrodes used for electrophysiological measurements in the brain are often similar in design to those required for electrochemical biosensing [16, 17]. Particularly, electrodes that ﻿are used for intracranial measurements in contact with the brain tissue in procedures such as electrocorticography (ECoG) and stereo-electroencephalography (sEEG) as opposed to those used for extracranial measurements on the surface of the skull, like those used for electroencephalography (EEG). These implantable electrodes are widely used in surgical (intraoperative) and pre-surgical (extraoperative) monitoring and evaluation of epilepsy with established procedures and reliability. Implantable depth electrodes used for sEEG typically have long flexible silicone shanks with many large platinum (Pt), iridium (Ir), or gold (Au) electrodes equally spaced along the shank length. Adapting a clinically approved platinum depth electrode for use in enzyme-based biosensors would simplify the route to testing and remove the need to develop and verify custom electrode devices. Enzyme-based electrochemical biosensors are one of the most established biosensing technologies with decades of work supporting their use in vitro and lately also in vivo in the form of wearable glucose biosensors [18–20]. These can be made with simple, robust functionalisation protocols that are easy to manufacture. Neurotransmitters such as L-glutamate, GABA, and acetylcholine can easily be measured using appropriate enzymes and electrode functionalisation protocols [21–24].

Here it is demonstrated that an existing clinically approved platinum depth electrode is electrochemically similar to standard platinum macro disc electrodes commonly used for electrochemical measurements. The deposition of a nanostructured platinum black coating on the electrode surface improves the surface area and increases the performance of the depth electrode but not to a point where it exceeds the performance of the pristine polished platinum polycrystalline disc electrode. The device was then modified and functionalised to provide a suitable platform for in vivo biosensing with in vitro testing showing reasonable sensitivity to either glucose or L-glutamate. This work is beneficial because it shows that an existing and approved medical device that is typically used for routine electrophysiology measurements and locating focal points for epileptic seizures can be easily adapted for the electrochemical detection of key neurotransmitters and metabolites.

## Materials and methods

A platinum (Pt) sEEG depth electrode (Spencer probe depth electrode, RD10R-SP07X-00, length: 390 mm, diameter: 0.86 mm, electrode length: 2.29 mm, electrode spacing: 7 mm, 10 cylindrical shank electrodes) was supplied courtesy of Glasgow University and Queen Elizabeth University Hospital, Glasgow (UK) and manufactured by Ad-Tech Medical, WI (USA). A Pt macro disc electrode, 1.6 mm dia., was purchased from ALS Co., Ltd. (Japan). A Pt foil counter electrode and an aqueous Ag/AgCl reference electrode filled with 3 M KCl were purchased from Metrohm, Herisau (Switzerland). Deionised (DI) water from an Elga LabWater PURELAB Chorus 2 lab water system was used to make all solutions. H_2_SO_4_ and H_2_O_2_ were purchased from Fisher Scientific (UK). PBS tablets, hexaammineruthenium(III) chloride, potassium ferricyanide, potassium ferrocyanide, gelatin powder, glutaraldehyde solution, dopamine hydrochloride, D-(+)-glucose, sodium L-glutamate, glucose oxidase from *Aspergillus niger*, and L-glutamate oxidase from *Streptomyces* sp. were purchased from Merck Sigma-Aldrich (UK). Chloroplatinic acid hexahydrate and lead acetate trihydrate were supplied courtesy of the Institute of Photonics, University of Strathclyde (UK).

Macro disc electrodes were manually polished using 1, 0.3, and 0.05 μm alumina particle and water slurry on a microfibre pad in a figure eight pattern for 60 repeats. Following each polishing particle size, the electrodes were rinsed with DI water and briefly placed in an ultrasonic cleaner in a beaker of DI water for 20 s then rinsed again. Electrodes were then electrochemically cleaned by applying 10 CV cycles in 0.1 M H_2_SO_4_ except where noted. Cleaning CV parameters: potential window −0.4 to +1.8 V, step 0.01 V, scan rate 0.1 V/s.

Phosphate buffer saline (PBS) solution, used as a biological buffer and electrolyte, was made at 1× concentration using PBS tablets in DI water as per the manufacturer’s instructions (1× is typically defined as a solution containing NaCl: 137 mM, KCl: 2.7 mM, Na_2_HPO_4_: 10 mM, KH_2_PO_4_: 1.8 mM, and is pH 7.4 at 25 °C). Solutions of 1 mM hexaammineruthenium(III) (HexRu) in 1× PBS and 1 mM ferricyanide and ferrocyanide (FF-Cy) in 1× PBS were used in all characterisation experiments. Experiments were performed in a 3-electrode cell with a working, counter, and reference electrode in electrolyte solution in an open beaker unless otherwise noted. Room temperature and standard atmospheric conditions were used except where noted. HexRu solutions were purged with argon for 10 min before use. The CV parameters used were 10 mV E-step, 3 scans, and 100 mV/s unless otherwise noted, and the potential window is shown in each experiment. The DPV parameters used were 10 mV E-step, 25 mV E-pulse, 0.05 s t-pulse, and 50 mV/s scan rate. The SWV parameters used were 10 mV E-step, 100 mV amplitude, and 20 Hz frequency. Electrochemical impedance spectroscopy (EIS) measurements were either taken at E^1/2^ for HexRu or at OCP measured for 10 s for FF-Cy, max freq. 100 kHz to min freq. 5 Hz in 44 steps, and 10 mV amplitude sine wave stimulation. EIS data was fitted to a Randles equivalent circuit model for extraction of parameter values.

Chronoamperometric (CA) measurements were taken with the working electrode at +0.7 V vs the Ag/AgCl reference electrode. CA measurements to characterise sensitivity were performed by stepwise additions of a stock concentration of analyte to a known volume and concentration, briefly stirred, allowed to settle for 100 s, and then the working electrode current value was extracted from the final data point before the next addition. For example, starting from a baseline of 10 min in 20 ml of 1× PBS, 20 μl of 100 mM glucose was then added, stirred, and allowed to settle for 6 repeated steps. This replicates the real-time nature of a recording in vivo and allows evaluation of the rise and settling time. Dopamine (DA) current measurements were extracted from the measured CVs at a potential of +0.25 V as this was approximately where the largest change was observed.

Electrodeposition of Pt black (also referred to in the literature as electroplating or platinization) was performed by applying a pulsed current rectangular waveform followed by a constant current waveform. The applied current of both the pulsed waveform and the constant current waveform was −0.3 mA. The pulsed waveform had a 1 s period and a 60% on duty cycle at the selected current and was 0 mA between the pulses. Sixty cycles of the pulsed waveform were applied followed by 60 s of constant current. The plating bath solution consisted of 7 mM chloroplatinic acid and 1 mM lead acetate in deionised water.

Dip coating solutions of 10 U/μl GOx, 0.5 U/μl L-gluOx, 1% w/v gelatin, and 1% glutaraldehyde all in 1× PBS were used unless otherwise noted. Functionalisation of the electrode and coating of the enzyme was performed by repeated dip coating in solutions of the selected enzyme, then gelatin, and then glutaraldehyde sequentially. Each dip coated solution was left to dry at room temperature for 3 min before the next was applied and this was repeated 5 times for a total of 15 dipped layers applied. The electrode was then left to finish crosslinking and dry for 16 h at room temperature before use. The dry crosslinked enzyme layer was rinsed, rehydrated, and stabilised in 1× PBS for 30 min before testing and use to wash out any free protein or crosslinker and minimise leaching or changes in the layer whilst recording measurements.

A PalmSens 4 potentiostat with MUX8-R2 multiplexer and PSTrace 5.9 software (PalmSens, Houten, Netherlands) was used for electrochemical measurements, data acquisition, baseline correction, and peak current measurements. OriginPro 2021b software (OriginLab, MA, USA) was used for all other data analysis and presentation. AFM images were acquired using an Asylum Oxford Instruments MFP-3D AFM with a Tap300Al-G probe, 40 N/m, 300 kHz, in AC air topography (tapping) mode.

## Results and discussion

### Electrode preparation

**Fig. 1:**
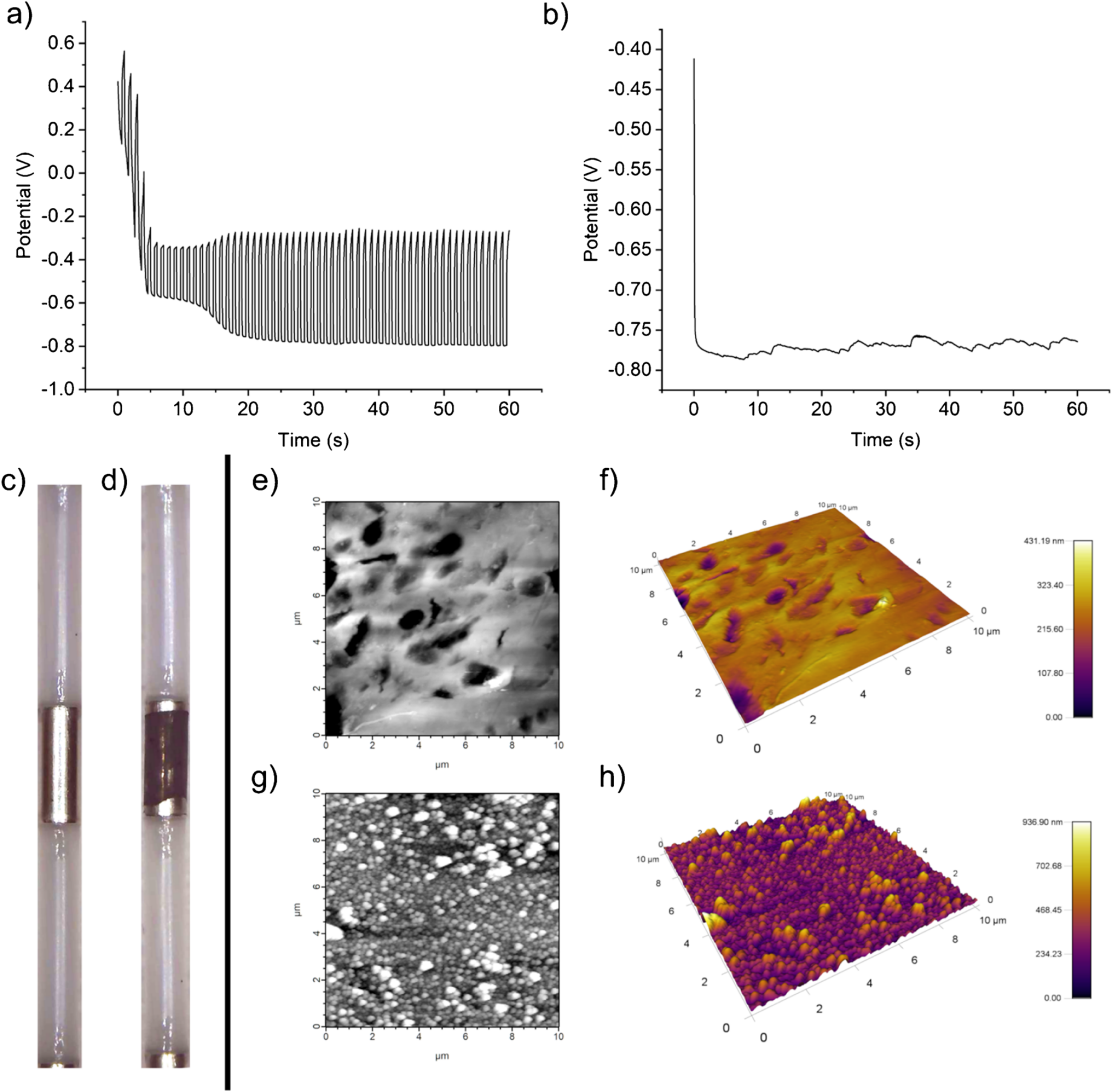
Platinum (Pt) black coating and electrode surface analysis. **a**, **b** Electrode potential measurement for the pulsed (**a**) and constant (**b**) current waveforms applied to the working electrode during electrodeposition showing stable growth and formation of the Pt black layer on the electrode surface. **c**, **d** Visual microscopy image of the electrode before (**c**) and after (**d**) Pt black electrodeposition. Inactive areas of the electrode and the roughly defined electrode shape are also visible after coating. **e**, **f** AFM 2D and 3D topography images of the depth electrode surface before Pt black electrodeposition. **g**, **h** AFM topography images of the electrode surface after Pt black electrodeposition

To enhance the performance of the Pt device, a roughened platinum black coating was electrodeposited on the depth electrode using a combination of a pulsed current rectangular waveform followed by a constant current waveform (Fig. 1a, b). This protocol resulted in the formation of a highly structured nanoscale surface that was porous and had a large active surface area (Fig. 1c, d). Surface area measured by integrating the hydrogen adsorption region of CVs in 0.1 M H_2_SO_4_ showed a substantial increase in active surface area from 13.2 mm^2^ before coating to 82.9 mm^2^ after coating. AFM measurements showed the average surface roughness increased with R_q_ increasing from 37 to 108 nm, and R_a_ increasing from 27 to 81 nm. Figure 1e, f show the electrode surface before coating and Fig. 1g, h show the surface after coating. Many small particles of approximately 200 nm in size can be seen in the AFM image and these are also likely to be highly porous which would account for the underestimate provided by the AFM roughness measurement when compared to the electrochemical analysis via CV. It can be concluded that the electrochemical treatment which was employed to produce a more electrochemically active surface through production of nanostructured Pt deposits was effective. The next task was to electrochemically characterise the depth electrodes with and without the platinum black coating and compare them against a pristine Pt polycrystalline macro electrode.

### Electrochemical characterisation of devices

**Fig. 2:**
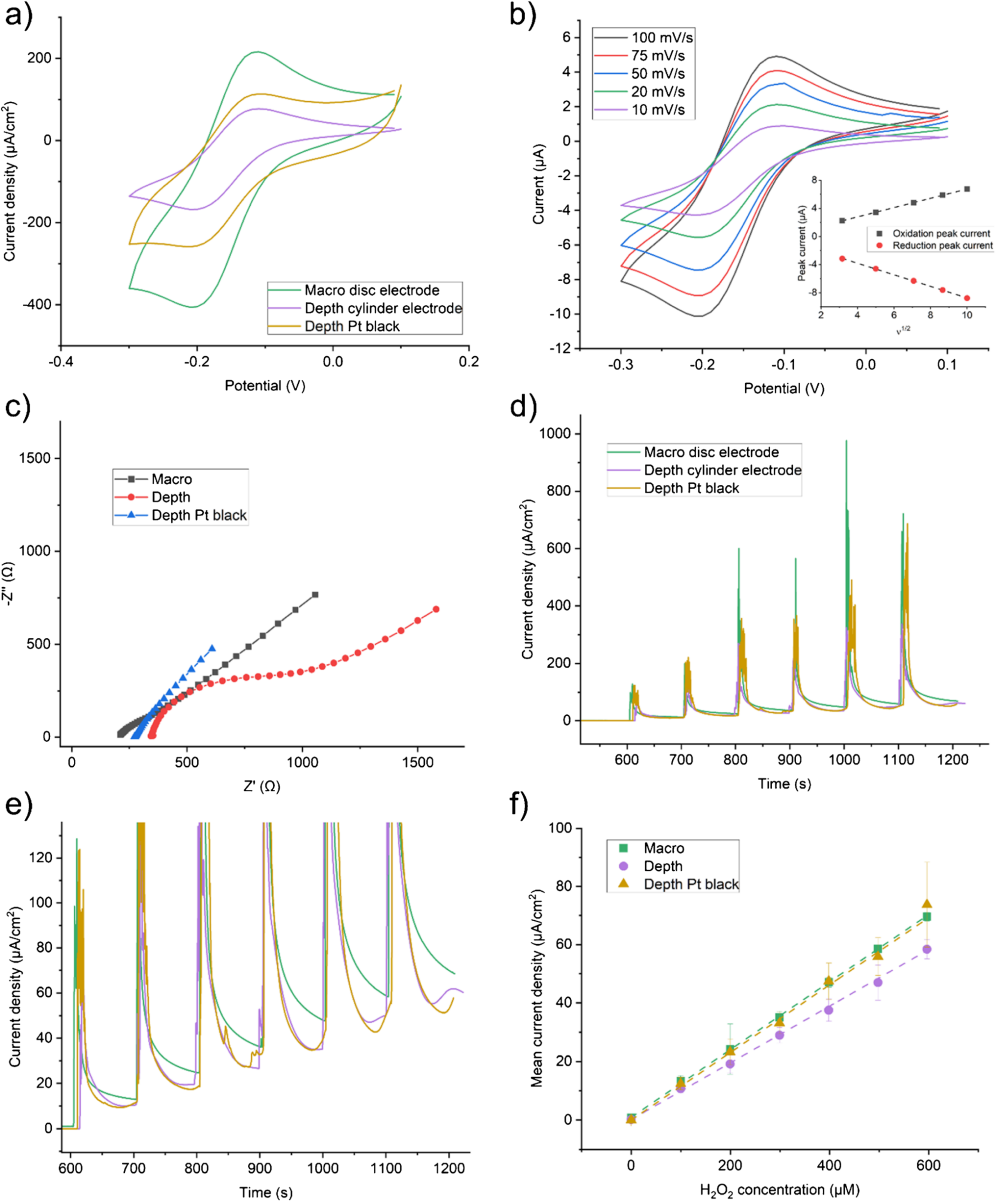
Electrochemical characterisation of the platinum (Pt) black coated sEEG depth electrode compared to the bare depth electrode and a standard Pt macro disc electrode. **a** CVs of 1 mM hexaammineruthenium(III) (HexRu) in 1× PBS purged with Argon showing the Pt black coated depth electrode to have comparable sensitivity to an ideal Pt macro disc electrode. **b** CVs of 1 mM HexRu in 1× PBS with increasing scan rate. Inset: Peak oxidation and reduction currents vs the SQRT of the scan rate (v^1/2^) and the linear fits showing the process is diffusion controlled and there is minimal adsorption. **c** EIS Nyquist plots in 1 mM ferri/ferro-cyanide (FF-Cy) in 1× PBS show that the Pt black coating reduces charge transfer resistance and increases double layer capacitance. Circuit fit data is given in Table 1. **d** Chronoamperometry (CA) of H_2_O_2_ in 1× PBS with repeated 20 μl additions of 100 mM H_2_O_2_ with the working electrode at +0.7 V vs Ag/AgCl aqueous reference electrode. **e** Enlarged view of the CA data and H_2_O_2_ addition steps shown in **d**. **f** Sensitivity calibration curve of measured current vs H_2_O_2_ concentration and respective linear fits. Linear fit *R*^2^ values: macro: 99.9, depth: 94.2, depth/Pt black: 99.8. Sensitivity and LOD data are given in Table 2

**Fig. 3:**
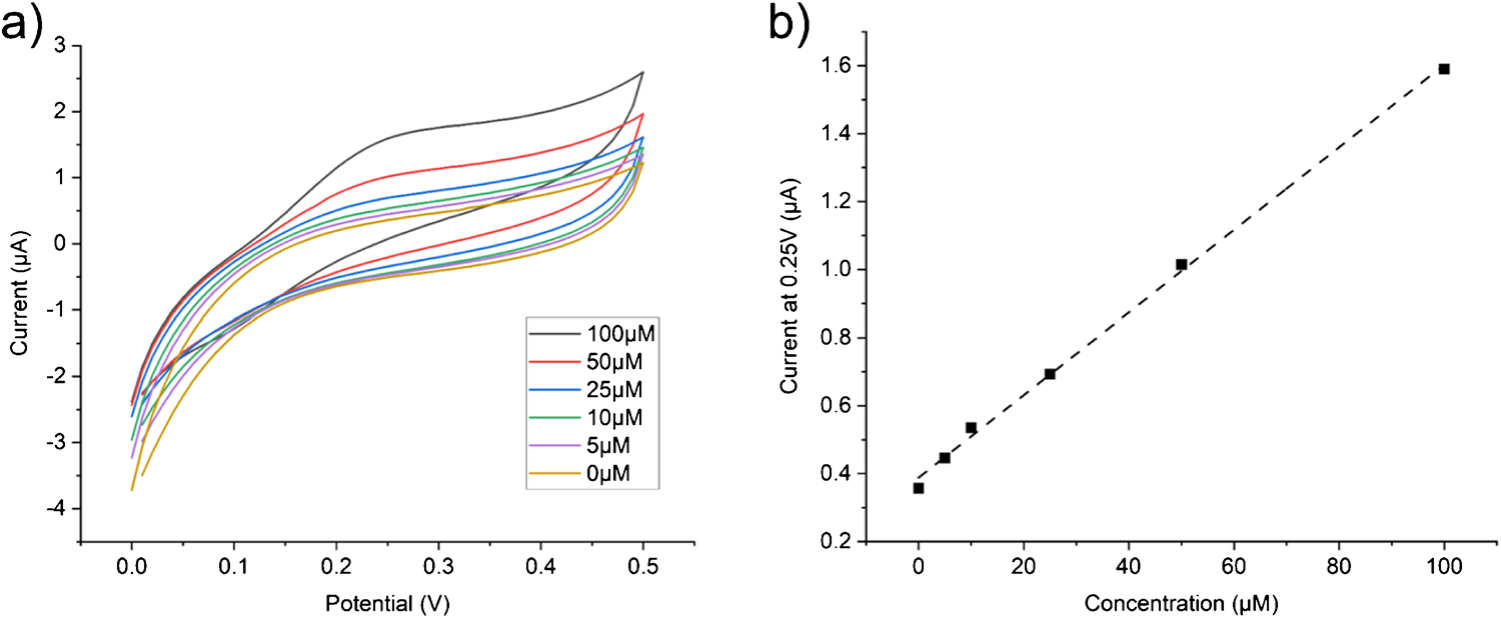
Dopamine (DA) measurements using the bare depth electrode as provided. DA concentration as noted in the plot. All DA solutions were made in 1× PBS. Three CV scans at 100 mV/s were measured and only the 3rd scan was used for analysis. **a** Representative CVs of increasing dopamine concentration. **b** CV current at +0.25 V vs DA concentration from the single electrode shown in **a**. The linear behaviour for this electrode is given as an estimate, sensitivity: 1.2 nA/μM, *R*^2^: 99.8

**Scheme 1:**
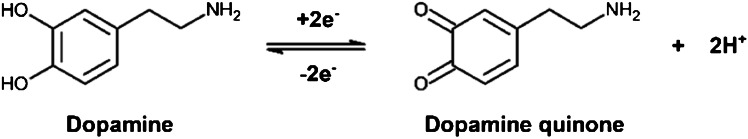
Electrochemical oxidation of dopamine to dopamine quinone takes place via a two-proton two-electron reaction

Having established a nano roughened platinum black coating, it was necessary to characterise the depth electrodes with and without coating and benchmark against a Pt macro electrode. Electrochemical characterisation of the platinum sEEG depth electrode using 1 mM HexRu showed the electrode was active as supplied without any preparation or cleaning. A polished and electrochemically cleaned macro disc electrode was included as a pristine model for comparison. Pt black coating further improved the sensitivity of the electrode. The peak potentials and currents were similar with the macro electrode having a slightly higher current density than the depth electrode. The Pt black coating improved the performance of the nano-modified depth electrode over the bare depth electrode; however, this was still not as sensitive as the pristine macro disc electrode (Fig. 2a). Plotting the HexRu reduction peak current measured on the unmodified electrode against the square root of the scan rate shows that they have a linear relationship which is associated with good linear diffusion and is the expected behaviour for a clean polycrystalline platinum electrode (Fig. 2b). EIS measurements in 1 mM FF-Cy (Fig. 2c) were fitted to a Randles circuit to extract equivalent circuit parameters and these are presented in Table 1. CA measurements of the current resulting from a given concentration of H_2_O_2_ in 1× PBS showed a clean linear response (Fig. 2d, e). The macro electrode was more sensitive than the depth electrode and again this was improved with use of the Pt black coating (Fig. 2f). Table 2 summarises the sensitivities and limits of detection (LOD) of the three electrode types compared. CVs of the bare Pt electrode in solutions containing a range of concentrations of DA also gave a linear current response (Fig. 3a, b). This is relevant to the in vivo measurements of neurotransmitters or as an interferent in other measurements. The DA reaction scheme is given in Scheme 1.

**Table 1:** EIS Randles circuit fit data for each type of electrode

Electrode type	Solution resistance Rs (Ω)	Charge transfer resistance Rct (Ω)	Double layer capacitance Cdl (mF)	Warburg (*σ*)
Macro disc	212.6	80.74	6.451E-4	4406
Depth	351.8	509.4	8.335E-4	4141
Depth/Pt black	277.4	7.005E-4	1.328E-2	2935

**Table 2:** Sensitivity and LOD for chronoamperometry measurements of H_2_O_2_ for each electrode type

Electrode type	Sensitivity (nA/cm^2^/μM)	LOD (μM/cm^2^)
Macro disc	115.67	11.71515
Depth	96.78	7.24125
Depth/Pt black	115.41	0.00234

Altogether, these results show that the out-of-the-packet depth electrode response was inferior to the response seen when the electrodes were modified with the platinum black coating. However, the platinum black modified electrodes were not able to reach the performance level of polished and electrochemically cleaned polycrystalline macro electrodes. Polishing and cleaning of the depth electrodes is not possible due to their form factor, and the visual and AFM imaging (Fig. 1c–f) shows that the electrode surface is rough and poorly defined. This is unsurprising because the depth electrodes are produced in a mass manufacturing environment and sealed for eventual use in the hospital. In these production conditions, it is not possible to have precise control of the electrode surface condition, and this is not a priority for electrophysiological measurements. Whilst the platinum black treatment was encouraging in that it produced improved results compared to unmodified depth electrodes, future work would involve developing the optimal surface conditions on the platinum depth electrodes to maximise electrochemical signal gain when measuring neurotransmitter chemicals in vivo*.* Having developed a platinum black coating approach that gave an enhanced signal, it was then necessary to coat the electrodes with a combination of gelatin-oxidase enzyme-glutaraldehyde and assess performance with the relevant analytes.

### Performance of enzymatically modified Pt depth electrodes

**Scheme 2:**
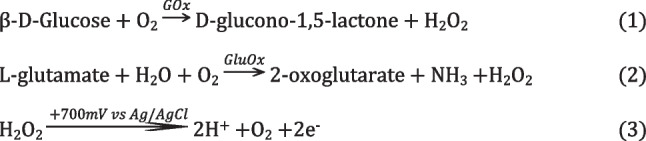
Enzyme catalysed reactions of glucose (1) and L-glutamate (2) producing H_2_O_2_ and the oxidation reaction of H_2_O_2_ with the working electrode in a two-electron process (3)

Functionalisation of the electrode with a simplified method for applying a glucose oxidase and gelatin-based coating allowed detection and measurement of glucose concentrations in a 1× PBS solution. The main target of interest was L-glutamate, to be detected via L-glutamate oxidase, but glucose oxidase served as a suitable test bed due to its higher activity levels and higher stability. The reaction schemes for glucose with glucose oxidase and L-glutamate with L-glutamate oxidase are given in Eqs. (1) and (2), respectively. The reaction scheme for H_2_O_2_ at the working electrode is shown in Eq. (3) (Scheme 2).

1 $$\upbeta -\textrm{D}-\textrm{Glucose}+{\textrm{O}}_2\overset{GOx}{\to}\textrm{D}-\textrm{glucono}-1,5-\textrm{lactone}+{\textrm{H}}_2{\textrm{O}}_2$$

2 $$\textrm{L}-\textrm{glutamate}+{\textrm{H}}_2\textrm{O}+{\textrm{O}}_2\overset{GluOx}{\to }2-\textrm{oxoglutarate}+{\textrm{NH}}_3+{\textrm{H}}_2{\textrm{O}}_2$$

3 $${\textrm{H}}_2{\textrm{O}}_2\overset{+700 mV\ vs\ Ag/ AgCl}{\to }2{\textrm{H}}^{+}+{\textrm{O}}_2+2\textrm{e}$$

**Fig. 4:**
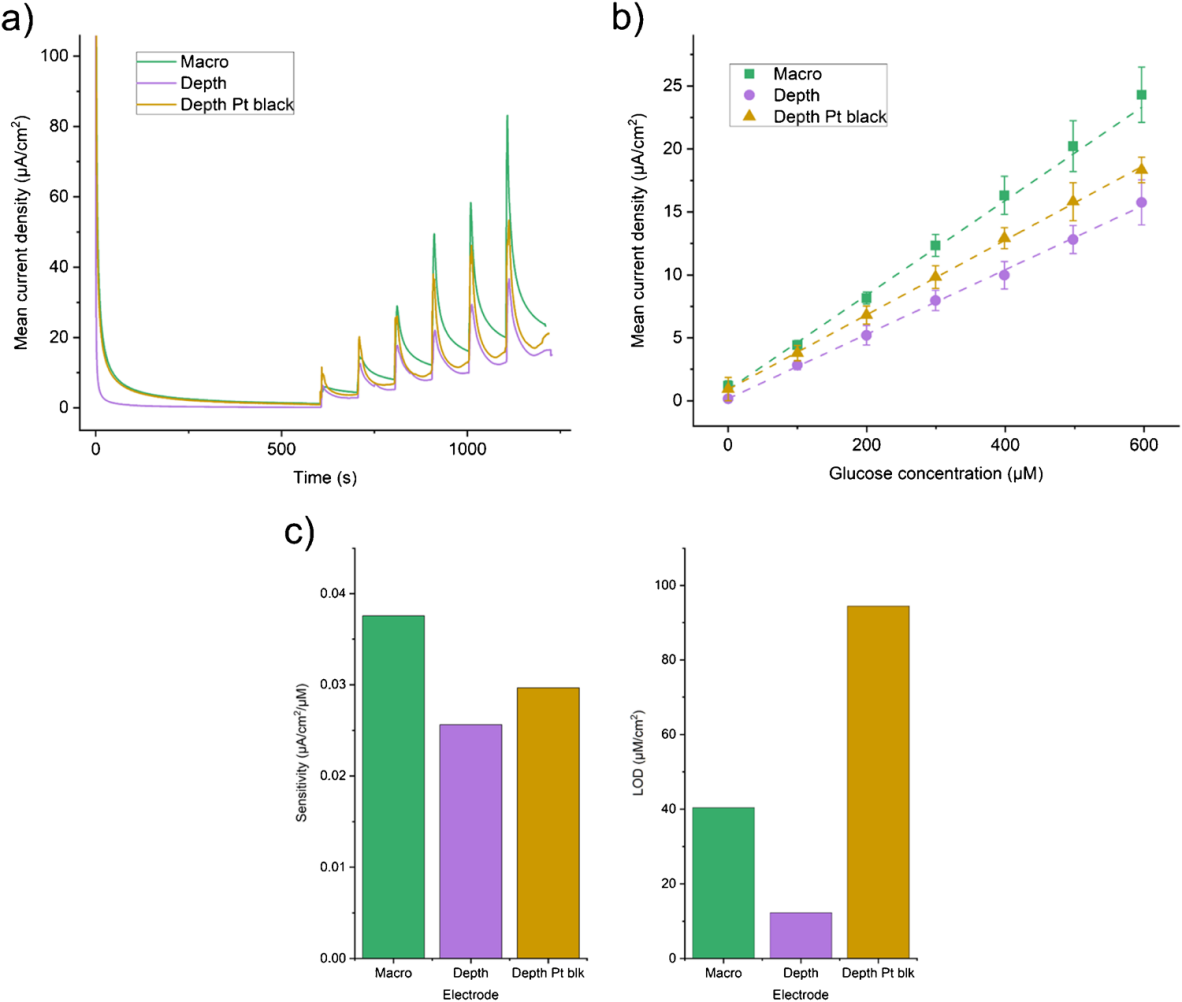
Glucose measurements in 1× PBS using the glucose oxidase enzyme coated electrodes. **a** Raw chronoamperometry data recorded with the working electrode at +0.7 V vs Ag/AgCl aqueous reference electrode. 10 Hz sampling frequency. Additions of 20 μl of 100 mM glucose start at 600 s and are repeated every 100 s thereafter. Oxidation current was recorded immediately before the following addition of glucose. **b** Calibration curves of the current vs the glucose concentration and the respective linear fits. *R*^2^ values for each electrode are macro: 99.6, depth: 99.9, depth/Pt black: 99.9 (*n* = 4). **c** Sensitivities from the slope of the linear fit to the calibration curve (left), and limits of detection (LOD) calculated using the 3 × SD of baseline method (right). Sensitivities in nA/cm^2^/μM; macro electrode: 37.57, depth electrode: 25.62, and depth Pt black electrode: 29.65. LOD in μM/cm^2^; macro electrode: 40.35, depth electrode: 12.23, depth Pt black electrode: 94.45

**Fig. 5:**
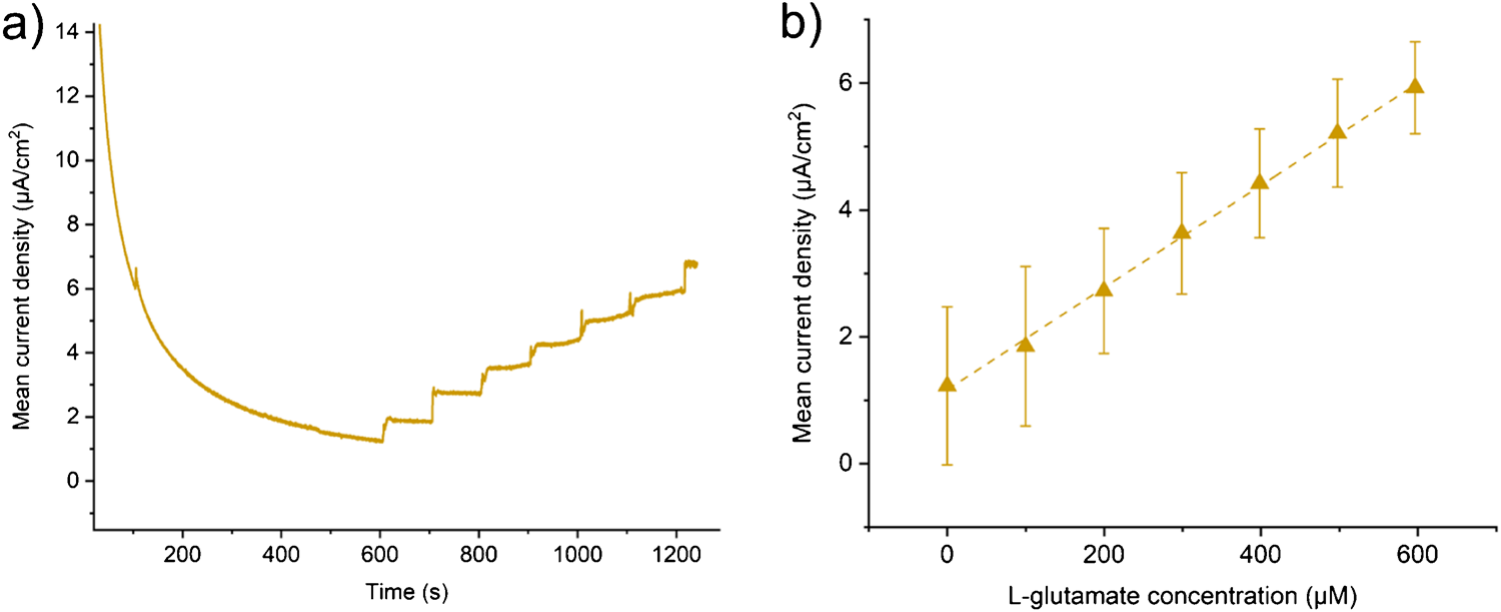
L-glutamate measurements in 1× PBS using the L-glutamate oxidase enzyme coated electrode. **a** Raw chronoamperometry data recorded with the working electrode at +0.7 V vs Ag/AgCl aqueous reference electrode. 10 Hz sampling frequency. Additions of 20 μl of 100 mM L-glutamate start at 600 s and are repeated every 100 s thereafter. Oxidation current was recorded immediately before the following additions of L-glutamate. **b** Calibration curve of the current vs the L-glutamate concentration and the respective linear fit. *R*^2^: 99.8 (*n* = 4). Sensitivity: 8.05 nA/cm^2^/μM, LOD: 471.7 μM/cm^2^

Figure 4a shows initial results with this enzyme coating. The calibration and sensitivity curve of current measured against concentration is shown in Fig. 4b. Figure 4c compares the sensitivity and LOD of each electrode type tested. Variability in the manufacturing and application of the enzyme layer is expected to play a large part in the measured sensitivity and LOD values. Replacing the glucose oxidase with L-glutamate oxidase allowed measurement of L-glutamate in an identical manner as shown in Fig. 5a. Both Figs. 4a and 5a show typical responses to the addition of analyte (a current spike) which eventually decays to reach a steady-state behaviour. The sensitivity to L-glutamate (Fig. 5b) is lower than that measured for glucose due to the lower concentration of the enzyme stock available due to cost and the lower activity of the specific enzyme used. However, the sensitivity is still within a usable range for measurements of L-glutamate during epileptic seizures (~1–10 μM) and could easily be improved by loading additional enzyme or further optimising the coating protocol. Optimisation of enzymatic sensing of L-glutamate has been undertaken elsewhere in the literature [22, 25]. The variability in manufacturing of the enzyme layer manifests as a sizeable interelectrode error; however, individual devices can be calibrated separately. In future work, care should be taken when applying in vitro calibration to in vivo measurements as there are many possible sources of error with these methods [26].

Altogether, the results present a surface treatment methodology for improving the sensitivity of surgically employed platinum sEEG depth electrodes and how these devices could be adapted from an existing clinically approved implantable device for electrophysiology measurements to obtain the additional capability of measuring dopamine and L-glutamate. Electrochemical characterisation and a simple enzymatic functionalisation of the electrode proved its suitability as a platform for further exploration and possible in vivo biosensing. When compared to other developed systems for human implantation, it can be seen that the most common examples in the literature are currently glass pulled carbon fibre or microfabricated silicon shank electrode devices that cannot be implanted to any great depth (beyond a few cm or mm respectively) and are not very flexible. This approach of modifying existing clinical sEEG depth electrodes has potential for combining electrophysiological and electrochemical measurements on a tested, easily manufactured, flexible platform. This could remove the need to develop novel electrodes as the technology is already in place and has been through the regulatory approval process, which critically involves sterility testing. Therefore, the reported approach has the potential to accelerate the time to clinical use of biosensors in the human brain. Excitingly, it means that existing electrochemical measurements of neurotransmission can be ported onto an existing platform. The findings presented here show that electrochemical measurements developed for traditional platinum surfaces translated easily onto the sEEG depth electrodes.

Personalised medicine needs targeted approaches to diagnostics and treatment of individual patients who may on the face of it have the same disease but upon further, more nuanced investigation may be found to have different modalities of disease or differently affected regions, e.g., in epilepsy, and the need to identify the focal point of seizures. Measurements of neurochemical fluctuations in the human brain could be an invaluable tool for treatment of a range of neurological diseases and disorders: Alzheimer’s, Parkinson’s, epilepsy and seizures, traumatic brain injuries, depression, and more [27–33]. Again, in terms of advances in personalised medicine, such devices could be chronically (permanently or semi-permanently) implanted for monitoring, diagnostics, treatment, and prevention of particular neurological conditions. This paradigm can also be extended to animals for research, understanding disorders, and developing possible treatments [34].

## Conclusion and outlook

This work has demonstrated that a clinically approved platinum sEEG depth electrode is suitable for use as a platform for in vivo enzyme-based biosensors. A nanostructured surface treatment and relatively simple enzymatic coating were applied so that L-glutamate could be measured at concentrations relevant to the detection of focal points of epileptic seizures. Using established electroanalytical approaches, the sEEG depth electrodes could directly measure dopamine in their unmodified form. Existing implantable electrode devices such as these, with a range of electrode configurations and materials, are readily available from several manufacturers and are clinically approved, widely understood, and supported by clinicians. As shown here, simple modifications and functionalisation protocols can be applied to the electrodes to produce biosensors for a range of analytes. This reduces the need to develop novel electrode platforms for in vivo biosensing and expands the potential for accelerated translation of novel neuro-electrochemical measurement approaches into clinical use.
